# Tetramethylpyrazine Induces Apoptosis and Inhibits Proliferation of Hypertrophic Scar-Derived Fibroblasts *via* Inhibiting the Phosphorylation of AKT

**DOI:** 10.3389/fphar.2020.00602

**Published:** 2020-05-05

**Authors:** Xue Wu, Zheng Wang, Gaofeng Wu, Xiaofan Xu, Jian Zhang, Yan Li, Hong Zhang, Shuzhen Guo

**Affiliations:** ^1^School of Traditional Chinese Medicine, Beijing University of Chinese Medicine, Beijing, China; ^2^Medical Experiment Center, Shaanxi University of Chinese Medicine, Xianyang, China; ^3^Shaanxi Collaborative Innovation Center of Chinese Medicinal Resources Industrialization, Shaanxi University of Chinese Medicine, Xianyang, China; ^4^The Key Laboratory of Biomedical Information Engineering of Ministry of Education, School of Life Science and Technology, Xi’an Jiaotong University, Xi’an, China; ^5^Department of Burns and Cutaneous Surgery, Xijing Hospital, The Fourth Military Medical University, Xi’an, China

**Keywords:** tetramethylpyrazine, hypertrophic scar-derived fibroblasts, apoptosis, hypertrophic scar, AKT

## Abstract

Hypertrophic scar (HS) is a serious fibrotic skin disease and often considered as a kind of benign skin tumor. Tetramethylpyrazine (TMP), the main chemical composition of the traditional Chinese medicine Chuanxiong Rhizoma, has shown significant clinical benefits in the treatment of fibrosis disease and tumor, while the role in HS and the concrete mechanisms remain elusive. Herein, the protective effects of TMP in the treatment of HS was investigated and the results showed that the protein expression levels of type I collagen (Col I), type III collagen (Col III), and α-smooth muscle actin (α-SMA) were all inhibited remarkably after addition of TMP in HS-derived fibroblasts (HFs). Moreover, TMP also suppressed fibroblast proliferative and induced cell apoptosis. The protein expression levels of Caspase-3 and Bcl-2 were all decreased comparing with the control group while proapoptotic proteins Bax and Cleaved Caspase-3 were increased. In addition, TMP treatment markedly reduced the phosphorylation levels of AKT. Taken together, our investigations demonstrated that TMP could down-regulate the expression of fibrosis-related molecules, inhibit scar fibroblast proliferation and activate cell apoptosis, during which AKT pathway was involved. Thus, this study shed more light on the pharmacological mechanisms of TMP, and provided a novel therapeutic alternative for prevention and treatment of HS.

## Introduction

HS is a serious skin fibrotic disease, often occurred after surgery, burn, trauma, and characterized by the excessive proliferation of fibroblasts and deposition of extracellular matrix proteins, mainly including Col I, Col III (Sidgwick and Bayat, 2012; Ikeda et al., 2013; Berman et al., 2017; Miletta et al., 2017). It was reported that fibroblasts and myofibroblasts were the main effectors in fibrogenesis (Djafarzadeh et al., 2013; Ikeda et al., 2013). Emerging evidence exhibited that there were more fibroblasts in HS tissues than that in normal skin. Furthermore, the synthesis of collagen I were increased in HS fibroblasts while the synthesis of collagenase were decreased. And subsequently, the ability to digest soluble collagen was reduced and the excessive collagen was synthesized and deposited, finally resulting in the formation of scar (Chiang et al., 2016; Hutchenreuther and Leask, 2016). Therefore, fibroblasts have become the potential therapeutic target for antifibrosis treatment (Wang et al., 2008; Honardoust et al., 2012; Hinz, 2016).

HS is usually considered as a benign skin tumor. Incidence rates of hypertrophic scarring vary from 40% to 70% following surgery to up to 91% following burn injury, depending on the depth of the wound (Gauglitz et al., 2011). Clinical treatments for HS include surgical resection, hormone therapy, laser therapy, RX oppression, and silicone gel therapy. However, the ability of these modalities to permanently eliminate HS is limited.

It has been generally recognized that traditional Chinese herbal medicines played a unique therapeutic role in the treatment of many diseases (Bhamra et al., 2017; Perry et al., 2018; Li et al., 2019). Tetramethylpyrazine (TMP), an effective component of the traditional Chinese medicine Chuanxiong Rhizoma, which has been used to treat cerebrovascular and cardiovascular diseases, pulmonary diseases and cancer (Huang et al., 2018; Shen et al., 2018). And it was reported that TMP could ameliorate fibrosis in myocardial pulmonary and liver (Wu et al., 2015; Wang et al., 2017; Zhao et al., 2017).

Our previous work revealed that TMP could inhibit the proliferation of fibroblasts and prevent the synthesis of collagens. These results were in accordance with the work reported in cardiac fibroblast (Nie et al., 2012). However, the detailed function and underlying molecular mechanism of TMP in HS therapy remain unknown. Therefore, in the present study, the effect and mechanism of TMP on collagen deposition, cell proliferation and apoptosis were investigated. Our research group have shown that AKT in hypertrophic scars was overactive, thereby activating its downstream signaling pathways, inhibiting cell apoptosis, and promoting cell proliferation and motility. In addition, the increased phosphorylation level of AKT could promote the development of hypertrophic scars (Shi et al., 2014; Shi et al., 2016). Moreover, inhibition of AKT increased apoptosis in murine HS model (Paterno et al., 2011).

For the significant role of PI3K/AKT pathways in cell proliferation and apoptosis, especially in the formation of HS reported in our previous work, PI3K/AKT pathways was enrolled in our work to elucidate the concrete molecular mechanisms of TMP on HS.

## Materials and Methods

### Cell Culture and Drug Treatment

All HS tissues was obtained from adult patients who were going to receive surgery in our department. The ages of patients range from 20 to 44 years old. Before the experiment, all patients were informed about the purpose and procedures of the study and voluntarily agreed to provide tissue. Written consent was obtained from all participants, and all protocols were approved by the Ethics Committee of Xijing Hospital, which is affiliated with the FMMU. All tissues were minced and incubated in a solution of collagenase type I (0.1 mg/ml; Sigma, St. Louis, MO, USA) at 37°C for 2.5 h to isolate fibroblasts. Fibroblasts were then pelleted and grown in Dulbecco’s Modified Eagle Medium (Gibco, Grand Island, NY, USA) supplemented with 10% fetal calf serum (Gibco), 100 U/ml penicillin, and 100 U/ml streptomycin. Cells were incubated at 37°C in a 5% (v/v) CO_2_-humidified atmosphere. All experiments were performed with passage 3–5 cells.

TMP was purchased from Shanghai Yuanye Biotechnology Co. Ltd with a purity of 98%. Ten milligrams of TMP was weighted and dissolved in 1 ml of DMSO to make up 10 mg/ml of TMP solution. Then, different volume of TMP solution was added to cells to make the final concentrations of 1, 5, 10, 20, 40 μM, respectively.

### Western Blot Analysis

HFs were seeded in six-well plates with a density of 2 ×10 5 cells per well and 70%–80% confluent HFs was starved for 12–16 h in serum-free medium. Then HFs were treated with different concentration of TMP for 48 h for cellular protein extraction. HFs were washed with ice-cold phosphate-buffered saline (PBS) and lysed using RIPA buffer supplemented with protease and phosphatase inhibitor mixtures (Heart Biological Technology Co. Ltd., Xi’ an, China) on ice. Lysates were separated by centrifugation at 4°C and 14,000 g for 10 min. Protein concentration was determined by BCA assay (Pierce, USA). Fifty milligrams total protein was subjected to sodium dodecyl sulfate-polyacrylamide gel electrophoresis (SDS-PAGE) and transferred to PVDF membranes (Millipore, Bedford, MA, USA). After blocking with 5% non-fat milk, the membranes were incubated with anti-alpha-smooth muscle actin (α-SMA) (1:200, Boster, China), anti-Col I (1:1000, Abcam, UK), anti- Col III (1:1000, Abcam, UK), anti-Bcl-2, anti-Bax, anti-Caspase-3, anti-Cleaved Caspase-3,anti-p-AKT, anti-AKT (1:1000, Cell signaling technology, USA), goat anti-Actin (1:200, Santa cruz biotechnology, USA) overnight at 4°C, next day the membranes were incubated with horseradish peroxidase conjugated secondary antibodies (1:3000) 37°C for 1 h. Then immunoreactive proteins were visualised using ECL western blotting detection reagent (Millipore, Billerica, MA) and detected using MultiImage Light Cabinet Filter Positions (Alpha Innotech, San Leandro, CA).

### Flow Cytometry Analysis

Cell cycle distribution was analyzed by flow cytometry (FACSAria, BD Biosciences). HFs (1 × 10 ^6^/ml) were treated with different concentration of TMP for 48 h according to the cell cycle distribution experiment. Then 48 h after treatment, cells were harvested, rinsed with PBS, fixed with 95% (v/v) ice cold ethanol and resuspended in staining buffer containing FITC Annexin V and propidium iodide (PI). The mixture was then incubated in the dark at room temperature for 15 min. The DNA contents of the stained nuclei were analyzed, and the number of cells in each cycle phase was calculated.

Cell apoptosis was detected using Annexin V-FITC Apoptosis Detection Kit I (BD Biosciences, San Diego, CA, USA) according to the manufacturer’s instruction. HFs(1 × 10 ^6^/ml) were treated with different concentration of TMP for 48 h and then digested with 0.25% trypsin, washed twice with cold PBS, resuspended in binding buffer and then incubated with Annexin V-FITC and PI for 15 min at room temperature in dark. Samples were then analyzed by FACS Calibur (BD Bioscience). The percentage of stained cells was determined using BD FACSDiva software (Becton, Dickinson, and company, Franklin Lakes, NJ, USA).

### CCK-8 Assay

The viability of HFs was detected by CCK-8 Cell Proliferation Assay Kit (Qihai, Shanghai, China) according to the manufacturer’s instructions. HFs were seeded into 96-well plates with a density of 5×10^3^ cells per well and treated with various concentrations (1, 5, 10, 20, and 40 µM) of TMP for 24, 48 and 72 h, respectively and then followed by incubation with CCK-8 solution for another 4 h. The amount of newly formed formazan dye was quantitated with a scanning multiwell spectrophotometer at 450 nm. The measured absorbance directly correlates with the number of viable cells.

### Statistical Analysis

Each experiment was repeated at least three times, and the data are presented as the means ± SD. Statistical differences between groups were analyzed by the Student’s t-test using SPSS 13.0. A P-value <0.05 was considered to indicate a statistically significant difference.

## Results

### TMP Treatment Inhibited Col I, Col III, and α-SMA Protein Expression in HFs

**Figure 1:**
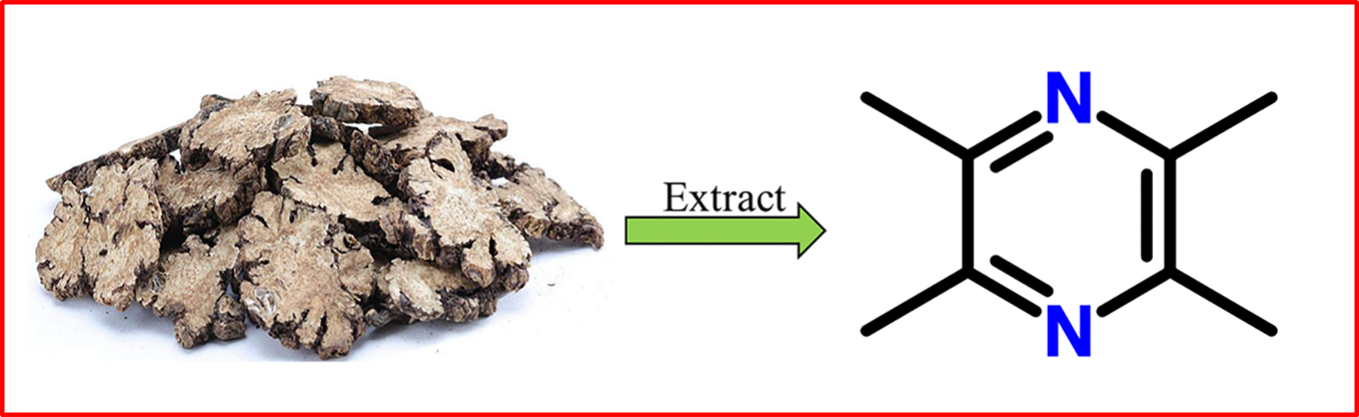
Chemical structure of TMP. TMP, tetramethylpyrazine.

**Figure 2:**
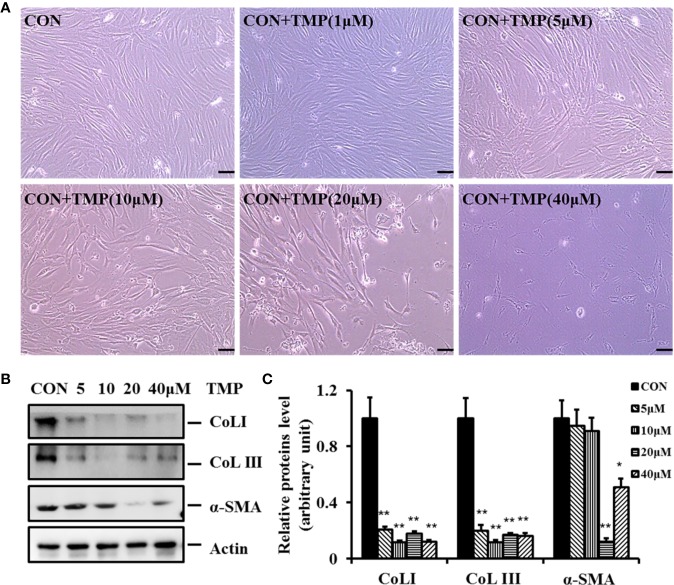
TMP inhibits the protein expression of Col I, Col III, and a -SMA in HFs. **(A)** Morphologies of HFs were visualized after different concentrations of TMP (0, 1, 5, 10, 20, and 40 µM) under light microscope. **(B)** HFs treated with different concentrations of TMP were starved for 12–16 h in serum-free DMEM, harvested after 48 h, and subjected to western blot analysis. **(C)** Histogram of the results. All values were normalized to either the negative control. Error bars represented means ± SD of n = 4. *p < 0.05 and **p < 0.01 vs. control group.

To demonstrate the inhibitory effect of TMP (chemical structure as shown in **Figure 1**) on HS, HFs were isolated and cultured in our experiment. HFs were treated with different concentrations of TMP (1, 5, 10, 20, and 40 µM) for 24 h and cell survival was then analyzed by light microscope. It was found that more cells presenting normal morphologies with 1 μM of TMP treatment. However, after addition of 5 and 10 μM of TMP, the cells became rounded, shrank, and floated. It was observed that the intercellular space increased and a large amount of cells swelled. Moreover, the cell number sharply decreased at 20 and 40 μM of TMP, and the cell morphology changed greatly (**Figure 2A**).

It is well known that the levels of fibrosis-related molecules such as Col I, Col III, and α-SMA are all increased in HS. In this study we found the protein expression levels of Col I, Col III, and α-SMA were simultaneously down regulated comparing with that in control group after treated with TMP (**Figures 2B, C**). Notably, TMP remarkably down-regulated Col I and Col III at the concentration of 10 µM. The protein expression level of α-SMA was greatly decreased after 20 µM of TMP treatment. These results revealed that TMP could decrease the expression and deposition of Col I, and Col III. Collectively, it was demonstrated that TMP may negatively regulate the expression of these fibrotic makers, but have no dose-dependent manner.

### TMP Inhibits HFs Proliferation

**Figure 3:**
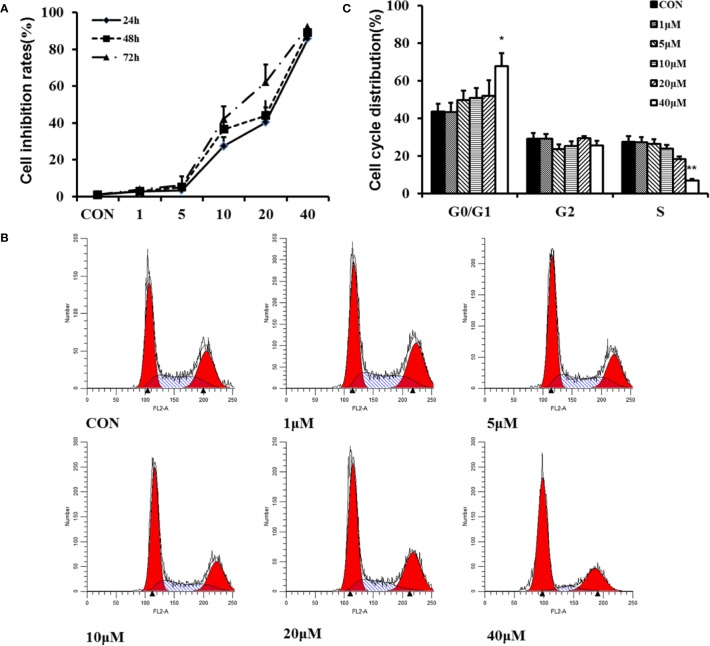
Effect of TMP on proliferation of HFs. **(A)** Cell proliferation was measured by the CCK-8 assay. HFs were seeded into 96-well plates at a density of 5×10^3^ cells per well and treated with various concentrations (1, 5, 10, 20, and 40 µM) of TMP for 24, 48, and 72 h, respectively. **(B)** Flow cytometry analysis showing the effect of TMP on cell cycle distribution. **(C)** Histogram summarized the results of **(B)**. Cell numbers at G1, G2, and S phases were counted and the percentage was calculated. Error bars represented means ± SD of n = 4. *p < 0.05and **p < 0.01 vs. control group. TMP, tetramethylpyrazine; HFs, HS-derived fibroblasts.

HFs excessive proliferation are important reasons leading to HS. Therefore, we determine the role of TMP in HFs proliferation. The results of CCK-8 experiment exhibited that HFs with various concentrations (1, 5, 10, 20, and 40 µM) of TMP for 24, 48, and 72 h inhibited the viability of cells (**Figure 3A**).

Flow cytometry analysis showed that TMP increased the number of cells in G0/G1 phase. Contrarily, the number of cells in the S phase was decreased along with the increased of the treatment concentration (**Figures 3B, C**). It was concluded that TMP could inhibit the proliferation of HFs through arresting the cell cycle at G0/G1 phase in a dose-dependent manner.

### TMP Induces Apoptosis and Regulates Apoptosis-Related Proteins Bcl-2 and Caspase-3 in HFs

**Figure 4:**
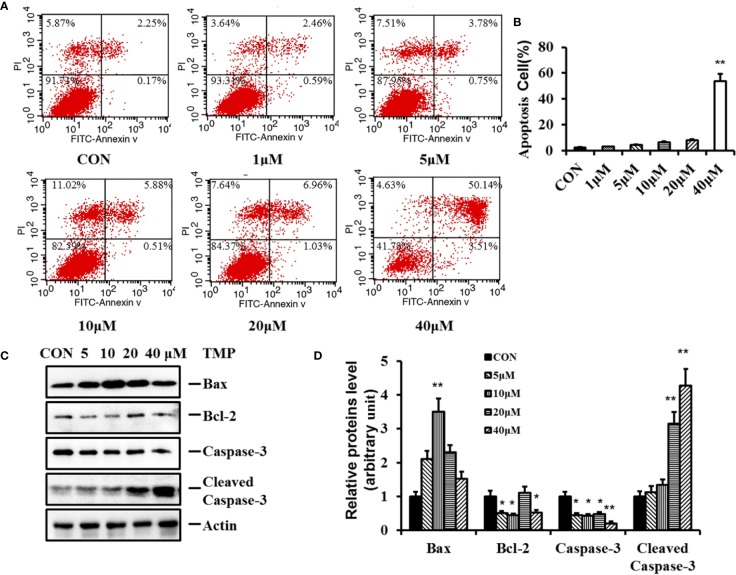
Effect of TMP on apoptosis of HFs and related protein Caspase-3 and Bcl-2. **(A)** HFs were treated with TMP at 1,5,10,20, and 40 µM for 48 h and cell apoptosis was analyzed by flow cytometry. **(B)** Histogram summarized the results of **(A)**. The activity of HFs was evaluated by Annexin-V/PI staining; **(C)** Western blot showed the protein level of Bax, Bcl-2, Caspase-3, and Cleaved Caspase-3 following the TMP treatment for 48 h. Actin served as an equal loading control. **(D)** Histogram summarized the results of **(C)**. Error bars represented means ± SD of n = 4. *p < 0.05 and **p < 0.01 vs. control group. TMP, tetramethylpyrazine; HFs, HS-derived fibroblasts.

To further assess the influence of TMP on HFs, cell apoptosis was investigated in our work. After treatment with TMP for 48 h, the number of both early and late apoptotic cells were increased in a dose-dependent manner. The treatment with 40 µM TMP induced early and late apoptosis in approximately 53.65% ± 1.56%. (**Figures 4A, B**).

Apoptosis is a complex and multistage process involving many genes and proteins. Caspase-3, Bcl-2, and Bax are three key proteins in the mitochondrial pathway inducing apoptosis. Caspase-3 is an early bio-markers of apoptosis activated through the proteolytic processing of procaspase-3 into 12 and 17 kDa subunits. Bcl-2 protein family serves as a crucial regulatory factor in the mitochondrial apoptotic pathway comprised of death inhibitors (Bcl-2, bcl-xL) and death activators (Bax, Bak). Bax is necessary for mitochondrial outer membrane permeabilization and it can be inhibited by the antiapoptotic protein Bcl-2. To further characterize the pro-apoptotic effect of TMP, the protein expressions of Bcl-2, Bax, Caspase-3 and Cleaved Caspase-3 were determined.The results in **Figure 4C** showed that after TMP treatment, the expression levels of Caspase-3 and Bcl-2 were all decreased, while the protein expression levels of Cleaved Caspase-3 and proapoptotic protein Bax were all increased comparing with control group. Furthermore, the protein expression ratio of Bcl-2/Bax was most significantly down regulated at 10 µM of TMP, while the protein expression ratio of Cleaved Caspase-3/Caspase-3 was most significant increased in 40 µM TMP group.

### Involvement of AKT Pathways in the Suppressive Effects of TMP in HFs

**Figure 5:**
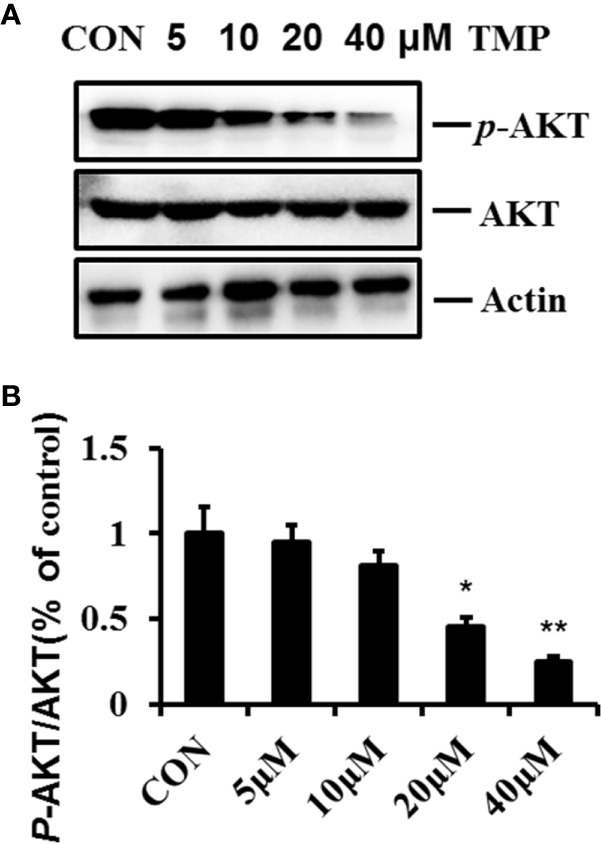
TMP inhibits the phosphorylation of AKT in HFs. **(A)** Cell lysates were analyzed by immunoblotting and quantified. Representative immunoblots of AKT and p-AKT (Ser473) were shown, with Actin as the loading control. **(B)** Histogram summarized the results of **(A)**. Error bars represented means ± SD of n = 4. *p < 0.05 and **p < 0.01 vs. control group. TMP, tetramethylpyrazine; HFs, HS-derived fibroblasts.

AKT pathway are related to the regulation of many cellular events, such as cell proliferation, survival and apoptosis. To gain mechanistic insight into the involved pro-apoptotic function of TMP, we analyzed the total and phosphorylation levels of AKT. The results showed that the ratio of p-AKT (Ser473)/total AKT were decreased following TMP treatment significantly in a dose-dependment manner and demonstrate that the AKT pathway has a crucial role in facilitating the pro-apoptotic effects of TMP(**Figures 5A, B**).

## Discussion

HS is the most commonly seen pathological scar, which leads to ache, pruritus, abnormal appearance and even limitation of motion (Aarabi et al., 2007).Currently, the treatment of pathological scar mainly includes pressure garment therapy, laser therapy as well as anti-scar drugs such as silicone gel (Honardoust et al., 2013; Miletta et al., 2017). However, there is no satisfying therapeutic approach available for hypertrophic scar treatment.

HS is characterized by excessive cell proliferation and ECM deposition (Honardoust et al., 2012; Honardoust et al., 2013), particularly Col I and Col III. Aberrant proliferation and apoptosis in fibroblasts, excessive ECM deposition often result in pathological scars (Hinz, 2007; Gauglitz et al., 2011; Saito et al., 2012). Thus, inhibiting over proliferation, activating fibroblasts apoptosis, and reducing collagen-based ECM deposition are highly important for HS treatment.

TMP has been demonstrated to regulate apoptosis in various cellular systems, and as a promising candidate for protection against fibrogenic insults (Zhang et al., 2014). However, the concrete molecular mechanism of these protective role of TMP on HS remain elusive.

In the current study, we found that pretreatment of HFs with TMP gave rise to cell morphological damages (**Figure 2A**). Furthermore, TMP significantly suppressed the protein levels of Col I, Col III, and α-SMA, but inconsistencies in Col I, Col III, and α-SMA changes at the same concentration of TMP. TMP remarkably down-regulated Col I and Col III at the concentration of 10 µM, while significantly down-regulated α-SMA at the concentration of 20 µM (**Figure 2B**). α-SMA is a well-known marker for myofibroblasts and is important for the contractile properties of myofibroblasts. Our results suggested the effects of the same concentration of TMP on collagen metabolism and the texture and contracture of scars were inconsistent in HFs. Overall, these results indicated TMP inhibitory effects on the overexpression of ECM in scar tissues and provided favorable aspects for TMP pretreatment in HS.

The dysregulation of dermal fibroblast proliferation and apoptosis is thought to be related to HS formation (Gauglitz et al., 2011). Thus, based on above findings, we were eager to elucidate whether TMP was correlated with its biological function in HS. By using CCK-8 and flow cytometry analysis, it was demonstrated that TMP could inhibit viability of cells (**Figure 3A**) and increase the number of cells in G0/G1 (**Figures 3B, C**), indicating that TMP suppressing fibroblast proliferation *via* redistributing cell cycle. The apoptotic cells that undergone both early and late apoptosis were determined by flow cytometry using Annexin V-FITC and PI staining, respectively. The results indicated that TMP could induce cell apoptosis of fibroblast (**Figure 4A**). Taken together, these results suggested that the possible inhibitory effects of TMP on HS were depended on inhibiting cell proliferation and promoting cell apoptosis.

Apoptosis is a complex, multistage process and it is worth noting that mitochondrial dysfunction is associated with cell apoptosis. Many studies have reported that the Bcl-2 family proteins regulated the mitochondrial apoptosis pathway and acted as sensors to integrate death and survival signals. Bax and Bcl-2 are two members of the Bcl-2 family. Moreover, caspase-3 activity has been described to be essential for drug-induced apoptosis, and Cleaved Caspase-3 is the active form of Caspase-3 which undergoes proteolytic processing at conserved aspartic residues to produce two subunits, large and small, that dimerize to form the active enzyme. In order to further verify apoptosis in HFs, we detected the protein expression levels of Caspase-3, Cleaved Caspase-3, Bax, and Bcl-2.

Our data demonstrated that TMP led to the up-regulation of Cleaved Caspase-3 and Bax, while the protein expression levels of Bcl-2 and Caspase-3 were down-regulated (**Figure 4D**). Interestingly, the protein ratio of Bcl-2/Bax with 10 µM of TMP treatment was smallest in all four investigated concentrations, indicating the most suitable concentration of TMP in regulating the balance of Bcl-2/Bax. However, the protein ratios of Cleaved caspase-3/caspase-3 were increased in a dose dependent manner. All our work implied that the inhibitory effect of TMP on fibroblasts may closely related with mitochondrial apoptotic pathway.

AKT pathway is known to be a major cell survival pathway and responsible for cell survival, energy metabolism and protein synthesis (Jiang et al., 2001; Castellino et al., 2009). Our previous studies confirmed that AKT in HS was overactive and deactivation of AKT signaling could inhibit the proliferation of fibroblast and excessive production of collagen in HFs (Wu et al., 2018). In addition, inhibition of AKT increased apoptosis in murine HS model. In this study, we found that AKT Ser473 phosphorylation level was gradually decreased within 5, 10, 20, 40µM of TMP treatment, suggesting AKT were involved in the protective effects of TMP on HS. This findings were consistent with previous research in which AKT was shown to be important in fibrosis diseases. Nonetheless, further investigations will be required to delineate the precise regulations of AKT signaling pathways in mediating TMP’s beneficial effects on HS.

## Conclusions

In conclusion, our study demonstrated that TMP pretreatment exerted remarkably protective effects on HFs *via* PI3 K/AKT signaling pathways. These findings contribute to our understanding of basic mechanisms regarding fibroblast development and scar formation. It may have future therapeutic implications for HS treatment.

## Data Availability Statement

All datasets generated for this study are included in the article/supplementary material.

## Ethics Statement

The studies involving human participants were reviewed and approved by Institutional Ethical Committee of the Fourth Military Medical University (FMMU). The patients/participants provided their written informed consent to participate in this study.

## Author Contributions

XW, GW, and XX designed and conducted the study, analyzed the data. XW and ZW wrote the manuscript. JZ and YL collected data. HZ and SG are the principal investigators, and revised and edited the manuscript. All authors approved the final manuscript.

## Funding

The present work was supported by the Program for the National Natural Science Foundation of China (NO. 81601693, 81703924, 81973687), the Foundation of Education Department of Shaanxi Province Government (NO. 18JK0212), the Key Research and Development Program of Shaanxi Province (2018ZDCXL-SF-01-02-02), the Youth Innovation Team of Shaanxi Universities.

## Conflict of Interest

The authors declare that the research was conducted in the absence of any commercial or financial relationships that could be construed as a potential conflict of interest.
